# How size matters: exploring the association between quality of mental health services and catchment area size

**DOI:** 10.1186/s12888-016-0992-5

**Published:** 2016-08-12

**Authors:** Taina Ala-Nikkola, Minna Sadeniemi, Minna Kaila, Samuli Saarni, Raija Kontio, Sami Pirkola, Grigori Joffe, Olli Oranta, Kristian Wahlbeck

**Affiliations:** 1 University of Helsinki and Helsinki University Hospital, Psychiatry, Välskärinkatu 12, FI-00029 Helsinki, Finland; 2 Mental Health Unit, National Institute for Health and Welfare (THL), Mannerheimintie 170, FI-00270 Helsinki, Finland; 3 Department of Social Services and Health Care, City of Helsinki, FI-00099 Helsinki, Finland; 4 Department of Public Health, Faculty of Medicine, University of Helsinki and Helsinki University Hospital, FI-000014 Helsinki, Finland; 5 Turku University Central Hospital and University of Turku, Kiinanmyllynkatu 4-8, 20520 Turku, Finland; 6 Department of Psychiatry, University of Helsinki and Helsinki University Hospital, Välskärinkatu 12, FI-00029 Helsinki, Finland; 7 University of Tampere and Mental Health Unit, National Institute for Health and Welfare (THL), Mannerheimintie 170, FI-33014 Tampere, Finland; 8 University of Helsinki and Helsinki University Hospital, Stenbäckinkatu 9, FI-00029 Helsinki, Finland

**Keywords:** Mental health services, Service providers, Diversity, Public corporation, Third sector, Private sector

## Abstract

**Background:**

The diversity of mental health and substance abuse services (MHS) available to service users is seen as an indicator of the quality of the service system. In most countries MHS are provided by a mix of public, private and third sector providers. In Finland, officially, the municipalities are responsible for organizing the services needed, but the real extent and roles of private and third sector service providers are not known.

Our previous study showed that the catchment area population size was strongly associated with diversity of mental health services. It is not known whether this was due to some types of services or some provider types being more sensitive to the size effect than others.

The aim of this study was to investigate the association between area population size and diversity of mental health services, i.e. which types of services and which service providers’ contributions are sensitive to population size.

**Methods:**

To map and classify services, we used the ESMS-R. The diversity of services was defined as the count of main types of care. Providers were classified as public, private or third sectors.

**Results:**

The diversity of outpatient, residential and voluntary services correlated positively with catchment area population size. The strongest positive correlation between the size of population and services available was found in third sector activities followed by public providers, but no correlation was found for diversity of private services.

The third sector and public corporations each provided 44 % of the service units.

Third sector providers produced all self-help services and most of the day care services. Third sector and private companies provided a significant part (59 %) of the residential care service units.

**Conclusions:**

Significant positive correlations were found between size of catchment area population and diversity of residential, outpatient and voluntary services, indicating that these services concentrate on areas with larger population bases. The third sector seems to significantly complement the public sector in providing different services.

Thus the third sector be needs to be functionally integrated with other MHS services to achieve a diversified and integrated service system.

**Electronic supplementary material:**

The online version of this article (doi:10.1186/s12888-016-0992-5) contains supplementary material, which is available to authorized users.

## Background

According to the World Health Organization (WHO) Mental Health Action Plan [1], provision of comprehensive, integrated and responsive mental health and social care services in community-based settings is a core aim.

On a more practical level, this aim relates to questions about the balance between community- and hospital-based mental health services and how to organize high quality services that are easily accessible to patients in the community [2–5]. Important components of community mental health services are multifaceted outpatient and day care services, mobile clinics, residential care in the community, outreach services, rehabilitation services, case management, information services, home treatment, supported employment, self-help and user groups and other ancillary services [6, 7]. Available evidence indicates that community-based and diversified mental health systems, with a wide range of services, are superior to hospital-centred mental health systems, according to a range of outcomes [8]. Thus availability of a diversity of mental health services can be seen as an indicator for the quality of the mental health service provision.

In many countries, non-governmental organizations (NGOs or third sector providers) have a significant role in supporting and providing easily accessible mental health and substance abuse services (MHS) [6, 9]. The third sector providers are often innovative in developing new services, or supplementing inadequate public infrastructure for mental health care [6]. The organizational structures of voluntary associations are often more nimble and adaptive than public agencies and professional associations, so they are predisposed to react quickly to environmental demands [6]. For example, private and voluntary providers of residential care have been shown to support different clientele than the public sector [10]. Thus they are often important providers of services that complement public services, increasing the diversity of services available.

In Finland the municipalities (*N* = 317, median population 5600) are responsible for organizing primary and secondary health care and social services. The municipalities can either provide the services by themselves or buy services from other providers, i.e. private companies, other municipalities or third sector organizations. To provide specialized health care services, the municipalities form hospital districts (*N* = 20, median population 169 652) [11–13]. On a yearly basis, the municipalities provide 2.4 million out-patient mental health visits. In addition to the tax-funded publicly organized health care system, there are private health care services and occupational health services which are partly supported by public funding (via the Social Insurance Institution). Occupational health services, which are available only for employed people only, provide approximately 50 000 visits to mental health professionals each year, and their role is thus very limited. Third sector organizations provide services as defined by public health care purchasers and voluntary services, which are supported by public funding from the Finnish Slot Machine Association game monopoly.

There is an increasing consensus in Finland that municipalities are by far too small to be responsible for organizing services, and far too many to provide coordinated integrated care with equal access. These problems are emphasized in MHS services, where clients often have multiple needs that can only be met by well-integrated (or even coordinated) care.

Integration of different mental health and substance abuse services has been a part of health policy since 2010, with little results so far [5, 13]. One hindrance to progress in integration of public health and social care has been that Finland is a very sparsely populated country (16 inhabitants/sq km). This means that increasing the size of catchment area populations may lead to huge increases in distance to services. Larger catchment areas are feared to lead to services being concentrated to central cities, further worsening access to services in more remote areas.

Thus one of the core issues to solve is the optimal size of population needed to arrange good enough horizontal and vertical integration of health and social care services or, in the context of this study, mental health services [13]. This is especially important because, as we found in our previous study, variation in catchment area population size explained 84 % of the variation in diversity (taken as indicator of quality) of MHS [14]. Diversity of services did not correlate with relative staff resources (full-time equivalents per citizens), nor with service needs of the population. Our previous findings suggested that planning and providing modern, diversified MHS requires catchment areas of at least 150 000 inhabitants, possibly even up to 500 000 [14]. However, as it is in practice impossible to simply increase the population base up to 500 000 inhabitants in Finland without increasing the distance to centralized services to hundreds of kilometers, it is essential to determine what kind of services are especially sensitive to catchment area size. This would allow the organizing of some services on a larger scale, whereas some services can be coordinated in smaller catchment areas.

A confounding factor not previously investigated is the effect different types of service providers might have on service diversity. Private companies and third sector providers might be located in more urban areas with more demand, thus mediating and emphasizing the effect of catchment area size on service diversity [9, 15]. This would be important to know, as regulating private and third sector providers is very different from regulating public providers.

The aim of this study was to investigate factors that could explain the association between population size and diversity of services. More specifically, to explore:

1. Whether some types of mental health and substance abuse services (MHS) are more sensitive to variations in population size
2. Whether some types of provider organization (public, private or third sector) are more sensitive to variations in population size

## Methods

### The study area

The data collection has been described previously [14]. Briefly, the study area included four hospital districts in the southernmost part of Finland. In total, the study area covers 2.3 million people, with 1.8 million adults (aged ≥18 years), and represents 43 % of the Finnish population. The study area is much more densely populated (averaging 174 inhabitants per square kilometre) than the whole country (average 16 inhabitants per square kilometre).

The four hospital districts consist of 67 municipalities, and are further divided into the 13 non-overlapping catchment areas: Länsi-Uusimaa (1), Lohja (2), Hyvinkää (3), Porvoo (4), Helsinki (5), Jorvi (6), Peijas (7), Carea (Kymenlaakso) (8), Eksote (South Karelia) (9), Turku (10), Salo (11), Vakka-Suomi (12) and Turunmaa (13). The adult population size varied within areas from approximately 18 200 (Turunmaa) to 500 000 inhabitants (Helsinki) (median 128 039, SD 122 759). All 13 areas have their own psychiatric hospital care, with some coordination at hospital district level. Psychiatric hospital care is integrated in general hospitals in some areas, but most areas still have free-standing psychiatric hospitals. The service mapping covers all adult (18+) mental health and substance abuse services in primary, secondary and tertiary health care, social services for people with mental disorders located in the catchment areas. Occupational health services could not be mapped, but only rarely provide specialist mental health services. Likewise, private psychiatrists and private psychotherapists could not be included, as they could not be reliably identified in the Finnish system. The study did not involve human subjects, so no ethics committee approval or written informed consent was needed.

### The instrument and data collection

The data collection and instrument have been described previously [14, 16]. Briefly, the instrument used for data collection was the Refinement Mapping Services Toolkit (REMAST) [17, 18]. It allows a description of the socioeconomic profile of the population of a specified area, alongside key features of mental health service provision, including those provided by primary care and social services. The service mapping was performed with the revised European Service Mapping Schedule (ESMS-R). The ESMS-R toolkit is derived from the previous European Service Mapping Schedule (ESMS) [19] and the Description and Evaluation of Services and Directories in Europe for Long-Term Care (DESDE-LTC) coding system [20]. The ESMS was designed to investigate MHS structures, describe their major characteristics, provision of services and resource allocation [19, 21, 22]. The previous version of the ESMS has been used in Finland [23] and other European countries [24–26].

Information was collected systematically by four researchers, who received special training for the ESMS-R. The data from three hospital districts was collected in 2012 and from the district of South-West Finland in 2013. Coding reliability was supported by service mapping procedure, case-based mapping training and duplication of coding by other raters and by a standardized handbook [18]. Data was collected using public data sources, as well as interviews with local health and social care representatives of municipalities and private providers. Sociodemographic data was derived from the SOTKAnet Statistics and Indicator Bank. Two thousand eleven data was used for the three districts, and 2012 data for the South-West Finland district (Additional file 1: Table S1).

### Measures and classification

In ESMS-R, mental health services are classified into 89 different “Main Types of Care” (MTC). MTC is the main descriptor of the care function (e.g. mobile acute team or acute hospital care). The MTC are organized in “Basic Stable Inputs of Care” (BSIC), i.e. the organizational units that provide the services (e.g. acute ward or day care centre). ESMS-R allocates MTCs into six main branches of care: 1) “Self-help and voluntary care”: facilities which provide for users support, self-help or contact, with unpaid staff, that offers accessibility, information, outpatient, day and residential care (as described in other branches); 2) “Outpatient care”: facilities which involve contact between staff and users for some purpose related to management of their condition and its associated clinical and social difficulties, that are not provided as a part of delivery of residential or day and structured activity services; 3) “Day care”: facilities which are normally available to several users at a time, providing a structured activity, or social contact and/or support, and expect service users to stay at the facilities beyond the periods during which they have face-to-face contact with staff; 4) “Residential care”: facilities which provide beds overnight for users for a purpose related to the clinical and social management of their health condition - users do not make use of such services simply because they are homeless or unable to reach home; 5) “Information for care”; facilities whose main aim is to provide users from the defined target group with information and/or an assessment of their needs. This service does not entail subsequent monitoring/follow-up or direct care provision; 6) “Accessibility to care”; facilities whose main aim is to facilitate accessibility to care for users with long-term care needs. These services do not entail direct care provision [17, 18].

Data on services in only five ESMS-R main branches were used for the final analyses, as no “Accessibility to care” services were found.

The legal status of each service provider was classified according to the Remast toolkit, which differentiates between six different legal categories: 1) Registered Charity, 2) Foundation, 3) Cooperative, 4) Social Firm, 5) Public Corporation and 6) Private Company [17]. In Finland, national legislation does not acknowledge the legal entity Registered Charities. The third sector in Finland consists mainly of associations and foundations [27, 28]. It is evaluated that approximately 17 % of citizens over 20 years are participating in third sector activities in social and health care [28]. The classification of legal status was adapted to better suit the Finnish context and thus in this study consists of: 1) Third Sector (Associations, Foundations, Social Firms and Cooperatives), 2) Public Corporations and 3) Private Companies.

### Data analysis

The diversity of MHS was determined by counting different MTC types in each catchment area. Associations between diversity of services and population size were explored using scatterplots, Spearman’s rank correlation and linear regression. The R^2^ coefficient was used to indicate the proportion of the variance in diversity of services explained by variation in catchment area population size. This was done separately for the four main types of services in order to see which services are most sensitive to variation in population size, and for the three categories of service provider’s legal status.

## Results

In total, 986 BSICs were found, divided into five main branches. The most common BSICs were residential care (*N* = 335, 34 %) and outpatient units (*N* = 291, 30 %) followed by self-help and voluntary services providing in total 190 (19 %) units, and by 149 day care BSICs (15 %) and 21 (2 %) information BSICs. We excluded from final analyses information (2 %) and accessibility for services (0 %) categories due to the small numbers.

There were 0.30 to 0.82 BSICs per 1000 citizens in different catchment areas (mean 0.53, SD 0.16). The BSICs identified, divided by main service branches and legal status are shown in Table 1. The self-help and voluntary services were wholly produced by third sector organizations; the public sector does not include volunteer work in Finland.

**Table 1:** Number of care units (BSICs^a^) sorted by ESMP-R^b^ main type of care (MTC) branches by legal provider

Catchment Area	Size of Population	Self-Help and voluntary				Outpatient				Day Care				Residential				All branches
		Public Corporation	Third sector	Private	Sum	Public Corporation	Third sector	Private	Sum	Public Corporation	Third sector	Private	Sum	Public Corporation	Third sector	Private	Sum	Total^c^ BSIC
Länsi-Uusimaa (1)	35296	0	4	0	4	6	0	0	6	4	2	0	6	3	0	4	7	23
Lohja (2)	70379	0	8	0	8	10	2	0	12	1	2	0	3	5	2	9	16	40
Hyvinkää (3)	139734	0	23	0	23	18	1	0	19	5	6	0	11	18	7	16	41	94
Porvoo (4)	74611	0	9	0	9	12	0	0	12	2	5	0	7	3	6	6	15	43
Helsinki (5)	501929	0	27	0	27	47	6	1	54	8	22	0	30	39	29	4	72	192
Jorvi (6)	230005	0	15	0	15	15	3	1	19	2	6	1	9	12	4	9	25	69
Peijas (7)	187332	0	11	0	11	21	1	1	23	1	9	0	10	9	10	10	29	77
Carea (8)	143265	0	23	0	23	19	11	1	31	5	11	0	16	14	19	11	44	116
Eksote (9)	109379	0	18	0	18	18	1	0	19	9	6	1	16	7	5	8	20	73
Turku (10)	151616	0	17	0	17	18	11	2	31	3	9	1	13	11	6	11	28	93
Salo (11)	128039	0	23	0	23	30	8	2	40	13	5	1	19	8	5	8	21	105
Vakka-Suomi (12)	81392	0	9	0	9	18	1	0	19	4	4	1	9	3	1	7	11	48
Turunmaa (13)	18200	0	3	0	3	6	0	0	6	0	0	0	0	2	0	2	4	13
	1871178	0	190	0	190	238	45	8	291	57	87	5	149	136	94	105	335	986
Legal providers part % of each MTC Branch			100,0		100,0	81,8	15,5	2,7	100,0	38,3	58,4	3,4	100,0	40,6	28,1	31,3	100,0	100^c^
MTC branch (%) of total BSICs					19,3				29,5				15,1				34,0	100^c^

^a^ BSIC = Basic Stable Input of Care; i.e. the organizational units that provide the services

^b^ European Service Mapping Schedule-reviSed (ESMS-R)

^c^ Information services (*N* = 21, 2 %) and Internet and State Hospital units (*n* = 3) are not shown but counted to the total units

The number of BSICs and MTCs provided by public corporations, third sector and private companies (Table 2). Public corporations provided in total 436 BSICs followed by third sector (430) and private companies (120).

**Table 2:** Number of care units (BSICs^a^) and different MTC^b^ sorted by providers legal status

Catchment Area	Size of catchment area population	Public Corporation		Third Sector		Private Company		Different MTC
		BSIC	Different MTC	BSIC	Different MTC	BSIC	Different MTC	
Länsi-Uusimaa (1)	35296	13	11	6	4	4	2	16
Lohja (2)	70379	17	8	14	7	9	3	16
Hyvinkää (3)	139734	41	13	37	15	16	2	26
Porvoo (4)	74611	17	8	20	7	6	2	13
Helsinki (5)	501929	94	16	93	26	5	3	38
Jorvi (6)	230005	29	16	29	13	11	5	27
Peijas (7)	187332	34	15	32	11	11	3	23
Carea (8)	143265	38	13	66	18	12	4	24
Eksote (9)	109379	34	16	30	8	9	4	23
Turku (10)	151616	34	12	44	19	15	6	32
Turku (10)	128039	52	17	42	15	11	7	21
Vakka-Suomi (12)	81392	25	8	14	5	9	6	19
Turunmaa (13)	18200	8	3	3	1	2	2	6
Total BSICs^c^	1871178	436		430		120		
%		44 %		44 %		12 %		
Different MTC			41		52		14	65
%			63 %		80 %		22 %	100 %

^a^ BSIC = Basic Stable Input of Care; i.e. the organizational units that provide the services, total BSICs are in Table 1

^b^ Different MTC (main types of care) in ESMS-R (European Service Mapping Schedule-reviSed) branches (*N* = 89)

^c^ Information services (*N* = 21, 2 %) and Internet and State Hospital units (*N* = 3) are not shown but counted to the total units

### Correlation between diversity and the size of population base

**Fig. 1:**
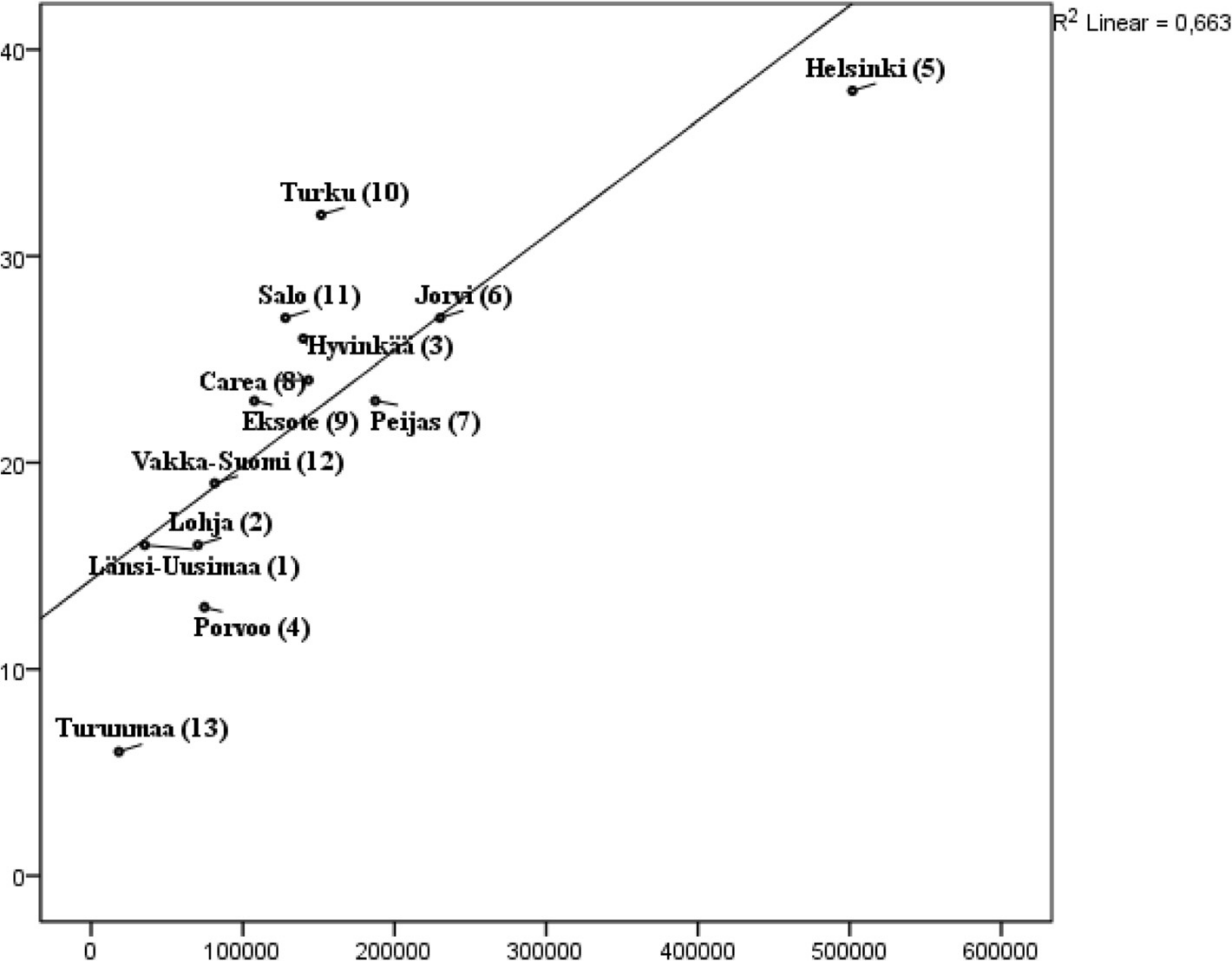
Association of the number of different MTC by all providers and size of population

Altogether there were 65 different MTC recognized out of 89 possible in the study area. The number of different types of services (MTC) available in the catchment areas varied significantly: from 6 to 38 (mean 22.3, SD 8.39) types. The largest diversity of services was found in the most populated area, i.e. Helsinki (38 different MTC), and the smallest diversity of services was in the smallest area, i.e. Turunmaa. There was a clear link between the catchment area population size and the diversity of services. The size of population explained 66.3 % of the variance in service diversity in the study area (Fig. 1).

### Correlation between the diversity of service types (MTC) and the size of the population base

**Fig. 2:**
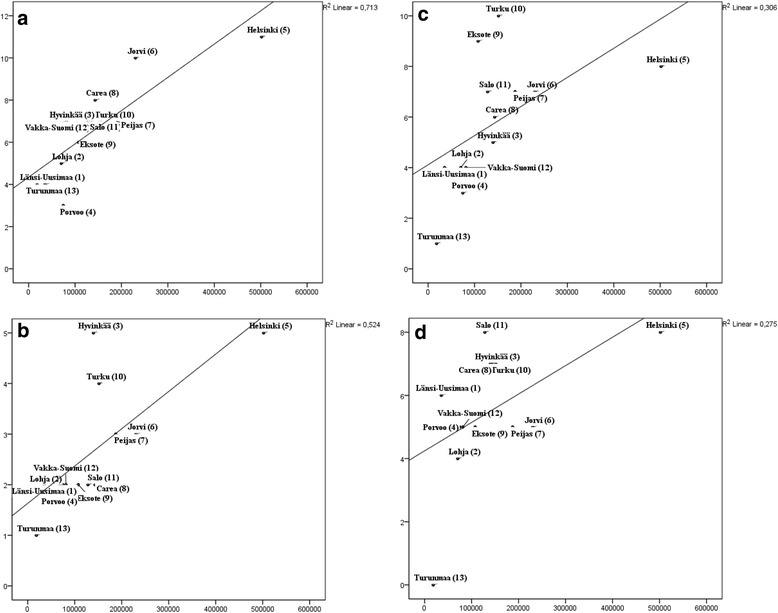
Associations of the number of different MTCs by: (**a**) residential, (**b**) self-help and voluntary, (**c**) outpatient, (**d**) day care and the size of population

Diversity of residential services was most strongly associated with population size (linear regression R^2^ 0.713), followed by self-help (R^2^ 0.524), outpatient services (R^2^ 0.36) and day care (R^2^ 0.275) services (Fig. 2).

The same order was also found using Spearman’s rank correlations. Diversity of services was statistically significantly correlated with catchment area size with high correlation coefficients (between 0.77 and 0.89, *p* <0.01) with the exception of day care services which did not quite reach statistical significance (0.547, *p* = 0.53).

The diversity of residential services correlated linearly with population, and most services were found in the most populated areas, i.e. Helsinki and Jorvi. Interestingly, the most diverse self-help and voluntary services were found in the capital Helsinki and in medium-sized areas Hyvinkää and Turku. Turku and Eksote boasted the most diverse outpatient services, followed by Helsinki, reflecting the local organization of mental health services.

### Correlation between the service provider and the size of population base

Public sector provided services classifiable to 41 different types of MTC. The third sector provided 52 different MTC and private companies 14 different MTC.

**Fig. 3:**
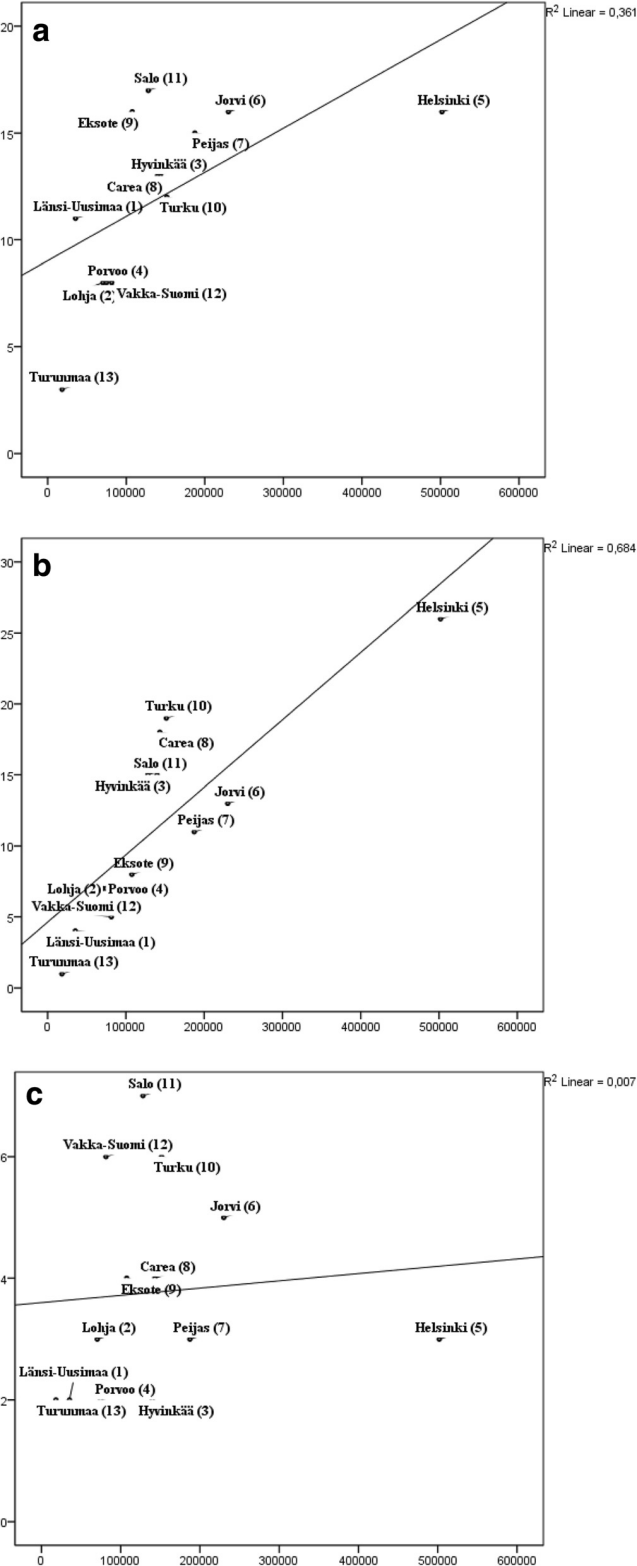
Associations of the number of different MTCs provided by: (**a**) public corporation, (**b**) third sector and (**c**) private sector, and the size of population

Correlations between population size and different MTC provided by public corporations, third sector and private companies are shown in Fig. 3a-c. The third sector services were clearly correlated with the population size (R^2^ 0.684), followed by services provided by the public sector (R^2^ 0.361). No association between population size and private services was observed. Similar results were obtained with Spearman rank correlations, where service diversity and population size correlated for public (0.715, *p* < 0.01) and third sector providers (0.848, *p* <0.01), but not for private providers (0.413, *p* = 0.161).

Of all BSICs identified, 44 % were owned by public corporations, 44 % by the third sector and 12 % by the private sector (Table 2). Public corporations provided most of the outpatient services (82 %), the third sector provided 100 % of self-help and voluntary services (Table 1). Residential service provision was quite evenly divided between different sectors, with public, private and third sector providing 41, 31 and 28 % of services, respectively. Day care services were mostly provided by the third sector (58 %) and public corporations (38 %).

The proportion of services provided by different sectors varied significantly between areas. The third sector provided between 23 and 55 % of service units, the private sector between 3 and 23 % of service units and the public sector between 33 and 62 % of service units.

## Discussion

We found that diversity of services was very clearly linked to catchment area size, with residential services and self-help and voluntary services showing the strongest link. The third sector services were strongly linked to catchment area size, whereas private services appeared to have no such link.

Non-governmental organizations are often thought to be significant actors in complementing and providing alternatives to existing public welfare services. A recent review of 127 articles on non-profit mental health organizations concluded that only very few (4 %) of non-profit organisations were seen as conflict-oriented or unprofessional, whereas most were concensus-oriented, aiming to collaborate with public services [29]. We are aware of little research regarding the geographical distribution of third sector mental health services, but some evidence exists that they tend to concentrate on more densely populate areas [29, 30]. Our results support these conclusions, in that mental health services by the third sector were mostly complementary to public services - especially the self-help and voluntary services not provided by the public system. But that was not the case in geographic sense, i.e. the third sector services were in our study concentrated on areas with larger population base, similarly to public services. A similar, although small, impact was found in an Australian study, where people in remote areas used slightly less non-professional services than those in urban areas [31]. Interestingly, in our study, it was the private services that did not show a link to catchment area size. This could suggest that the third sector services complement public services qualitatively, but private services complement the lack of other services geographically.

Our results can also be viewed in the light of general mental health policy directions and recommendations, especially the WHO Mental Health Action plan for 2013–2020 [1]. The plan underlines a multisectoral approach for a comprehensive and coordinated response for MHS with public sector and other providers, like third sector and private sector, as partners [1]. Thus the governance of MHS is not only public governance, but also extends to the relationship with third sector and private partners. Effective and accountable coordination between policies, regulation and leadership for MHS is needed to promote the mental health of the whole population. Resources for MHS are commonly limited, adding to the need for strong coordination between different sectors.

In practice, the fragmentation of psychiatric services, and the lack of coordination with social welfare or the labour administration, negatively afflicts many mental health care systems. The cost of under-, double- or over-provision of services caused by this phenomenon is estimated to be high [32]. Other negative consequences of fragmentation include treatment discontinuation and neglect of specific risk groups. Efforts to only address the consequences of fragmentation are often costly or have only limited effects. Ideally, public mental health services should focus on diminishing the detrimental effects of service fragmentation, typical to MHS services [32].

Our results represent the present reality in many places: MHS services are provided by a mixture of public, private and third sector providers. To reduce this potential for inequality, third sector providers could have a more active role in planning of services. Flannery et al. [7] argue for role clarity and note that outcomes of services are often dependent on outcomes of effective and appropriate community MHS. Thus the sustainability of third sector activities will also depend on effective collaboration and coordination between sectors [15]. The remarkable role of third sector providers, in our study, in complementing public services with different kinds of services suggests this coordination could be achieved with cooperation, not competetion. Adding some optimism is that, as Karlsson and Markström have convincingly shown [29], third sector actors are overwhelmingly oriented towards cooperation and collaboration with public services. This suggests that the public services should be active in searching for cooperation. On the other hand, the practical challenges of coordination are highlighted by our results: the third sector has a major role in providing community-based services – actually providing all self-help and a remarkable part of day care – and these services are concentrated in larger cities.

The whole-system approach that includes nationally coordinated public services, private and third sector has also been seen valuable for complex psychosis rehabilitation, where failure to develop one ideal type of support may be compensated by another provider or services [33]. The modern community based mental health care system calls for diverse outpatient services, but also diverse residential services [3, 4]. In our study more diverse residential services - alternatives for inpatient hospital care -, were located on more populated areas. This has been observed earlier for both population density and geographic distance on rural areas [26, 34].

### The results in Finnish context

Finland is a Nordic welfare state, where the main funding source for public health and social services is tax. In spite of the tax funding of public corporations, the third sector still provided 44 % of BSICs — as many as the public sector - and the third sector provision of services was more diversified than public services (52 and 41 different types of services, respectively). One reason for this might be, that also the third sector providers receive public funding from various sources. In addition, the number of BSICs does not directly show the economic part different sectors play. As the public service units are larger, the public sector still provides about three times as many work hours as the third sector (data not shown).

Another Finnish factor is that the country is relatively sparsely populated, and many of the areas investigated are relatively small in population. Long distances might hinder the practical possibilities in organizing diverse self-help and voluntary activities; the costs of outreach services increases and clients willingness to travel to centralized services decays, as the distances get longer. On the other hand, more populous areas in Finland also include the largest cities, which have the highest educated population. This might correlate with willingness to participate in third sector activities.

### Strengths and limitations

One strength of the study is the use of a standardized and internationally validated instrument (ESMS-R) for investigating service structure. The instrument allows for the coverage of services in primary, secondary and tertiary health care as well as social and the third sector. Our service mapping did not cover out-of-pocket private care, such as psychiatrists and psychologists in private practice, which constitute approximately 20 % of out-patient visits and is at least partly reimbursed by the social insurance institution. However, private services are all office-based and do not contribute to the diversity of the service system, because also public services offer such office-based outpatient services.

Only one per cent of visits in occupational health care are visits to mental health professionals, and thus the effect of missing out occupational health services in the service mapping is negligible. Furthermore, as occupational health services are office-based, their omission does not reduce the diversity of services because similar office-based outpatient services are offered in public health services.

The most important limitation of the study to note is, however, that service diversity is not the only indicator of service quality. The hypothesis here is, that a wide variety of specialized services is better than the opposite. Still, it is obvious that real health effects happening on individual level require many more successful steps along the process of care. Thus our next research line will be to correlate these organizational findings to health databases and public health outcomes.

Service diversity was strongly correlated with catchment area size. The mapping instructions on the ESMS-R require, that service units are mapped to only one main type of care. Thus it is possible, that some service diversity is lost from view due to the classification system, and this could be more marked on the smallest units, where specialization is most difficult. However, claiming that a small unit can provide a vide variety of different services without specializing is perhaps beside the point, which was to measure the diversity of clearly specialized services.

The small number of observations hindered the possibilities to conduct very detailed model diagnostics. The assumptions of the parametric methods were interpreted as satisfied as the correlation coefficient estimates based on the Pearson, Spearman and Kendall methods were close to each other’s (data not shown).

Lastly, our cross-sectional study only shows associations, but not causal relationships — which in the context of MHS structures are undoubtedly complex. For example, many factors associate with larger cities, like higher level of education and younger population (data not shown). In linear regression modelling the education years after primary school were significantly associated with the higher diversity of services provided by third sector. The relation between population size and service diversity is also unlikely to be direct, but mediated by many factors — most obviously the volume of services and resources available. For example, available BSICs and service diversity correlated significantly. Thus further research on longitudinal health outcomes data is very important, as planned in the EU-Refinement project, as would be qualitative studies investigating the actual processes that have led to creation of best-quality MHS systems.

## Conclusions

The study replicates on a larger scale our previous finding, that catchment area population size is strongly associated with diversity of MHS. Especially third sector provision was strongly associated with population size, as was diversity of residential services. Overall, the third sector’s role of MHS production was larger than expected in all types of services. For example, the third sector provides all self-help and voluntary services, almost all information services and most day care services. This makes rational planning and resourcing of MHS as a whole difficult.

In practice the results of our study suggest, firstly, that larger population bases are needed in order to provide diversified services, especially in residential services. Variation in service diversity across areas was large, indicating unequal access to services in different part of country. Secondly, any plans and processes to coordinate, improve and legislate MHS as a whole must include third sector services as an integral part of the package.

## Abbreviations

BSIC, Basic Stable Input of Care; DESDE-LTC, Description and Evaluation of Services and Directories in Europe for Long-Term Care; ESMS-R, European Service Mapping Schedule, Revised; MHS, Mental health and substance abuse services; MTC, Main types of care; NGO, Non-governmental organization

## Additional file

Additional file 1: Catchment areas background information. (XLSX 41 kb)
